# Prognostic factors after isolated ipsilateral local and regional recurrence in HER2-negative luminal breast cancer: a multi-center retrospective study

**DOI:** 10.1186/s12957-023-02991-1

**Published:** 2023-03-27

**Authors:** Yong Hwa Eom, Chang IK Yoon, Young Joon Kang, Ye Won Jeon

**Affiliations:** 1 Division of Breast Surgery, Department of Surgery, Eunpyeong St. Mary’s Hospital, College of Medicine, The Catholic University of Korea, Seoul, Korea; 2 Division of Breast Surgery, Department of Surgery, Seoul St. Mary’s Hospital, College of Medicine, The Catholic University of Korea, Seoul, Korea; 3 Division of Breast Surgery, Department of Surgery, Incheon St. Mary’s Hospital, College of Medicine, The Catholic University of Korea, Incheon, Korea; 4 Division of Breast Surgery, Department of Surgery, St. Vincent’s Hospital, College of Medicine, The Catholic University of Korea, Suwon-si, Gyeonggi-do, Korea

**Keywords:** Breast cancer, Ipsilateral, Local recurrence, Luminal, Prognosis

## Abstract

**Background:**

Although the incidence of isolated ipsilateral local and regional recurrence (IILRR) in human epidermal growth factor 2 (HER2)-negative luminal breast cancer is low, it is important because of its potential risk of distant metastasis and breast cancer related mortality. The aim of this study was to investigate prognostic factor and survival of IILRR using a large multi-center cohort.

**Methods:**

Data on patients with HER2-negative luminal breast cancer between 2005 and 2015 were retrieved. The endpoint was IILRR rate, post-recurrence progression-free survival (P-PFS), and post-recurrence overall survival (P-OS). Prognostic factors for progression and overall survival (OS) after IILRR were assessed by multivariate analysis.

**Results:**

Eighty (2.37%) patients experienced IILRR. Of them, 27 (33.7%) experienced a disease progression, including 23 (85.2%) who had distant metastasis. The median DFS was 48.5 months (range, 4–138 months). In 72.5% of cases, the first IILRR occurred after 3 years. Estimated 5-year P-PFS rates were 86.2%, 69.7%, 69.0%, 42.7%, and 82.2% for patients with age < 40 at diagnosis (*p* = 0.015), T1 stage (*p* = 0.012), stage I (*p* < 0.001), lymphovascular invasion (*p* = 0.003), and patients with post-recurrence endocrine therapy (*p* < 0.001), respectively. The 5-year Kaplan–Meier P-OS rate for patients was 81.4%. Post-recurrence endocrine therapy was independent factor for progression (HR: 0.176, *p* < 0.001) and OS (HR: 0.080, *p* < 0.001).

**Conclusions:**

Although there is no standardized treatment for IILRR yet, endocrine therapy after local resection plays a more important role in improving prognosis than chemotherapy or radiotherapy in HER2-negative luminal breast cancer.

## Background

Despite years of advances in a more extensive use of adjuvant systemic treatment, contemporary multidisciplinary approaches, and an understanding of the risk about locoregional recurrence (LRR) of breast cancer, patients and their physicians are unaware of LRR of breast cancer [1, 2]. The overall incidence of LRR has been reported to be 5–15% in breast cancer and 0.8 to 2.6% in hormone receptor (HR)-positive breast cancer [1–6]. Compared with LRR including breast, chest wall, axillary lymph nodes, internal mammary nodes, or supraclavicular lymph nodes, isolated ipsilateral local and regional recurrence (IILRR) occurring in breast, chest wall, and axillary lymph nodes are much less common [6–10]. IILRR is a rare event with an estimated rate of 0.5–1.5% per year after breast conserving surgery for invasive carcinoma and an overall incidence ranging from 5 to 10% after 10 years of follow-up [6–8].

Factors influencing IILRR include positive surgical margin, extensive intraductal component, vascular invasion, and tumor multicentricity as pathological characteristics. Others include young age at first diagnosis of breast cancer, HR status, lymph node status, tumor size, BRCA1/2 mutation carriers, radiotherapy, systemic treatment, and endocrine therapy [6, 9].

Despite the low rate of IILRR, the occurrence of isolated ipsilateral recurrence is important because it carries a substantial risk of distant metastasis and breast cancer-related mortality [5, 9, 11]. However, the incidence is so low that there are few studies on prognosis and survival after IILRR [11, 12]. In the case of human epidermal growth factor 2 (HER 2)-negative luminal breast cancer which is known to have a good prognosis, the incidence of IILRR is lower than that of other molecular subtypes. Thus, there are few studies on IILRR rate, prognostic, and survival after IILRR [13–15].

Therefore, the aim of this study was to investigate the progression and survival after IILRR in human epidermal growth factor 2 (HER2)-negative luminal breast cancer and to identify prognostic factors affecting the progression and survival after IILRR in a large multi-center cohort.

## Methods

### Cohorts of patients

In our cohort study, we searched women with primary nonmetastatic, HER2-negative luminal breast cancer diagnosed between January 2005 and December 2015 in four hospitals (Seoul St. Mary’s Hospital, St. Vincent’s Hospital, Incheon St. Mary's Hospital, and Eunpyeong St. Mary's Hospital) associated with the College of Medicine of the Catholic University of Korea.

Among these patients, we analyzed clinicopathologic data for eligible HER2-negative luminal breast cancer patients who were diagnosed with ipsilateral breast (or chest wall or skin) and/or axillary lymph nodes recurrence after initial curative surgery with axillary staging (axillary lymph node dissection(ALND) or sentinel lymph node biopsy(SLNB)). All breast cancer recurrences in the ipsilateral breast (or chest wall or skin) and/or axillary lymph nodes were pathologically confirmed after surgical resection. Patients with in situ (stage 0), metastatic breast cancer at diagnosis (stage IV), bilateral breast cancer, or breast cancer recurrence in internal mammary nodes or supraclavicular lymph nodes were excluded.

Patients’ demographics and tumor characteristics including age, type of surgery, pathological T and N staging, breast cancer stage according to the eighth edition of American Joint Committee on Cancer (AJCC) classification [16], histologic grade, type of adjuvant treatment (chemotherapy, endocrine therapy, and radiotherapy), Ki-67 status, estrogen receptor (ER), progesterone receptor (PR), HER2 expression, and type of post-recurrence treatment were reviewed. Their hormone receptor status was determined using an enzyme immunoassay. It was reported in the medical record. A positive ER and PR status was defined as an Allred score (AS) of ≥3.

Immunohistochemistry (IHC), fluorescence in situ hybridization (FISH), or silver in situ hybridization (SISH) was performed to evaluate the HER2 status. An IHC score of 0, +1, or an IHC score of +2 and FISH/SISH negativity was defined as negative for HER2 expression. The Ki-67 score ranged from 0 to 100%. Its positive cut-off level was ≥ 15 because this level was the median value in the entire cohort. The local ethics committee or institutional review board at each institution approved this study (approval number: XC22RIDI0030).

### Statistical analysis

IILRR was defined as the development of breast cancer in the ipsilateral breast (breast parenchyma after breast conserving surgery), chest wall (after total mastectomy), skin (after breast conserving surgery), or the ipsilateral axillary lymph node. Disease-free survival (DFS) was defined as the time from surgery to the time of first IILRR. Progression was defined as the development of subsequent loco-regional recurrence and/or distant metastasis.

Post-recurrence progression-free survival (P-PFS) was defined as the time from first IILRR to the date of progression (subsequent loco-regional recurrence and/or distant metastasis). Post-recurrence overall survival (P-OS) was calculated from the time of diagnosis of the first IILRR to the time of death from any cause.

The chi-square test and analysis of variance (ANOVA) were used for comparison between categorical variables and the two-sample *t*-test was used for comparison between continuous variables. Survival curves were estimated using the Kaplan–Meier method. Log-rank tests were performed for comparing survival curves. Multivariate analyses were conducted using Cox’s proportional hazard regression models to study effects of ipsilateral tumor recurrence on DFS and overall survival (OS). Parameters included in the multivariate analysis model were as follows: patient age, tumor size, lymph node status, AJCC classification, type of ipsilateral recurrence, and type of palliative treatment. All tests were two-sided and a *p*-value less than 0.05 was considered to be statistically significant. All analyses were performed using SPSS version 18.0 for Window (IBM Corp., Armonk, NY, USA).

## Results

### Patients with IILRR characteristics

Of 3373 patients of the overall cohort with HER2-negative luminal breast cancer, 80 met the eligibility criteria for this study. The incidence of IILRR was 2.37%. The median follow-up time from the date of initial operation was 111.5 months (range: 6–197 months).

Baseline characteristics of the analyzed population with IILRR are described in Table 1. The median DFS was 48.5 months (range: 4–138 months). IILRR occurred in 27.5% of cases within 3 years after initial operation and in 72.5% of cases after 3 years.

**Table 1:** Baseline characteristics of the analyzed population with IILRR

	IILRR (*n* = 80)	Percent (%)
Age (year) at diagnosis		
< 40	17	21.2
≥40	63	78.7
Breast operation		
Breast conserving surgery	49	61.2
Mastectomy	31	38.8
Axilla operation		
No	1	1.2
SLNB	28	35.0
ALND	51	63.8
pT		
1	47	58.7
2	33	41.3
pN		
0	57	71.2
1	17	21.3
2	4	5.0
3	2	2.5
Pathologic stage		
I	68	85.0
II	10	12.5
III	2	2.5
ER status		
Negative	2	2.5
Positive	78	97.5
PR status		
Negative	15	18.7
Positive	65	81.3
HG		
1	19	23.7
2	42	52.5
3	19	23.8
Ki-67		
≤15%	39	48.7
> 15	41	51.3
LVI		
No	49	61.2
Yes	30	37.5
Unknown	1	1.3
Adjuvant chemotherapy		
No	37	46.3
Yes	43	53.7
Adjuvant radiotherapy		
No	31	38.8
Yes	49	61.2
Adjuvant endocrine therapy		
No	5	6.2
Yes	75	93.8
Site of first recurrence		
Breast	46	57.5
Axilla	15	18.7
Chest wall	13	16.3
Skin	1	1.2
Breast + axilla	3	3.8
Axilla + chest wall	2	2.5
Number of recurrence		
One	75	93.7
Two or more	5	6.3
Post-recurrence chemotherapy		
No	45	56.2
Yes	35	43.8
Post-recurrence radiotherapy		
No	63	78.7
Yes	17	21.3
Post-recurrence endocrine therapy		
No	34	42.5
Yes	46	57.5
Progression		
No	46	57.5
Yes	27	33.7
Locoregional	4	14.8
Distant	23	85.2
Unknown	7	8.8
Death		
No	66	82.5
Yes	14	17.5

*SLNB* Sentinel lymph node biopsy, *ALND* Axillary lymph node dissection, *ER* Estrogen receptor, *PR* Progesterone receptor, *HG* Histologic grade, *LVI* Lymphovascular invasion

At the time of first diagnosis, 17 (21.2%) patients aged less than 40 years and 63 (78.7%) patients aged more than 40 years. Forty-nine (61.2%) patients underwent breast conserving surgery and 31 (38.8%) patients underwent mastectomy. Of the 80 patients who met the eligibility criteria of this study, most of them (*n* = 68, 85.0%) were at stage I. Forty-three (53.7%), 49 (61.2%), and 75 (93.8%) patients received chemotherapy, radiation therapy, and endocrine therapy after initial diagnosis, respectively.

Among 80 patients with IILRR, 49, 20, 15, and 1 patient had breast recurrence, axilla recurrence, chest wall recurrence, and skin recurrence, respectively. Of 80 patients, 75 (93.7%) had one site of recurrence and 5 (6.3%) patients had two sites of recurrence at the same time. After the first recurrence, progression occurred in 27 (33.7%) patients. Fourteen (17.5%) of 80 patients died.

### Prognostic factors for progression after IILRR

Of the 80 patients with ipsilateral recurrence, 7 had follow-up loss after ipsilateral recurrence. Thus, data from 73 patients were analyzed (Table 2). After the first ipsilateral recurrence, 27 (33.7%) patients experienced progression. The median time from the first ipsilateral recurrence to progression was 45.0 months (range: 1–136 months). After IILRR, progression rate was 65.8% within 5 years and was 34.2% after 5 years.

**Table 2:** Clinical and pathological characteristics according to progression in patients with recurrence

Characteristics	No (*n* = 46) (%)	Yes (*n* = 27) (%)	*p *value
Age (year) at diagnosis			0.022
< 40	14 (30.4)	2 (7.4)	
≥40	32 (69.6)	25 (92.6)	
Breast operation			0.528
Breast conserving surgery	29 (63.0)	15 (55.6)	
Mastectomy	17 (37.0)	12 (44.4)	
Axilla operation			0.014
No	1 (2.2)	0	
SLNB	20 (43.5)	4 (14.8)	
ALND	25 (54.3)	23 (85.2)	
pT			0.007
1	32 (69.6)	10 (37.0)	
2	14 (30.4)	17 (63.0)	
pN			0.206
0	35 (76.1)	17 (63.0)	
1	8 (17.4)	7 (25.9)	
2	3 (6.5)	1 (3.7)	
3	0	2 (7.4)	
Pathologic stage			0.007
I	43 (93.5)	18 (66.7)	
II	3 (6.5)	7 (25.9)	
III	0	2 (7.4)	
ER status			1.000
Negative	1 (2.2)	1 (3.7)	
Positive	45 (97.8)	26 (96.3)	
PR status			0.384
Negative	8 (17.4)	7 (25.9)	
Positive	38 (82.6)	20 (74.1)	
HG			0.095
1	15 (32.6)	3 (11.1)	
2	22 (47.8)	15 (55.6)	
3	9 (19.6)	9 (33.3)	
Ki-67			0.319
≤15%	26 (56.5)	12 (44.4)	
> 15	20 (43.5)	15 (55.6)	
LVI			0.012
No	33 (71.7)	11 (40.7)	
Yes	12 (26.1)	16 (59.3)	
Unknown	1 (2.2)	0	
Adjuvant chemotherapy			0.646
No	23 (50.0)	12 (44.4)	
Yes	23 (50.0)	15 (55.6)	
Adjuvant radiotherapy			0.412
No	16 (34.8)	12 (44.4)	
Yes	30 (65.2)	15 (55.6)	
Adjuvant endocrine therapy			1.000
No	2 (4.3)	1 (3.7)	
Yes	44 (95.7)	26 (96.3)	
Site of first recurrence			0.101
Breast	30 (65.2)	10 (37.0)	
Axilla	7 (15.2)	7 (25.9)	
Chest wall or skin	7 (15.2)	7 (25.9)	
Breast + axilla or axilla + chest wall	2 (4.3)	3 (11.1)	
Number of recurrence			0.352
One	44 (95.7)	24 (88.9)	
Two or more	2 (4.3)	3 (11.1)	
Post-recurrence chemotherapy			0.138
No	27 (58.7)	11 (40.7)	
Yes	19 (41.3)	16 (59.3)	
Post-recurrence radiotherapy			0.869
No	35 (76.1)	21 (77.8)	
Yes	11 (23.9)	6 (22.2)	
Post-recurrence endocrine therapy			<0.001
No	9 (19.6)	18 (66.7)	
Yes	37 (80.4)	9 (33.3)	
Selective estrogen receptor modulators	15 (40.5)	7 (77.8)	
Aromatase inhibitors	22 (59.5)	2 (22.2)	
Death			<0.001
No	46 (100)	13 (48.1)	
Yes	0	14 (51.9)	

*SLNB* Sentinel lymph node biopsy, *ALND* Axillary lymph node dissection, *ER* Estrogen receptor, *PR* Progesterone receptor, *HG* Histologic grade, *LVI* lLymphovascular invasion

Only 4 (14.8%) patients had progressed LRR. Twenty-three (85.2%) patients had distant recurrence as further progression event (Table 1). Patients with progression after the first ipsilateral recurrence were associated with age of 40 years or older, higher pathologic stage, lymphovascular invasion (LVI), and receiving post-recurrence endocrine therapy (Table 2). Factors associated with the risk of progression after the first ipsilateral recurrence in univariate analysis were age at diagnosis, initial pathologic stage including T and N stage, LVI, and post-recurrence endocrine therapy (Table 2).

**Fig. 1:**
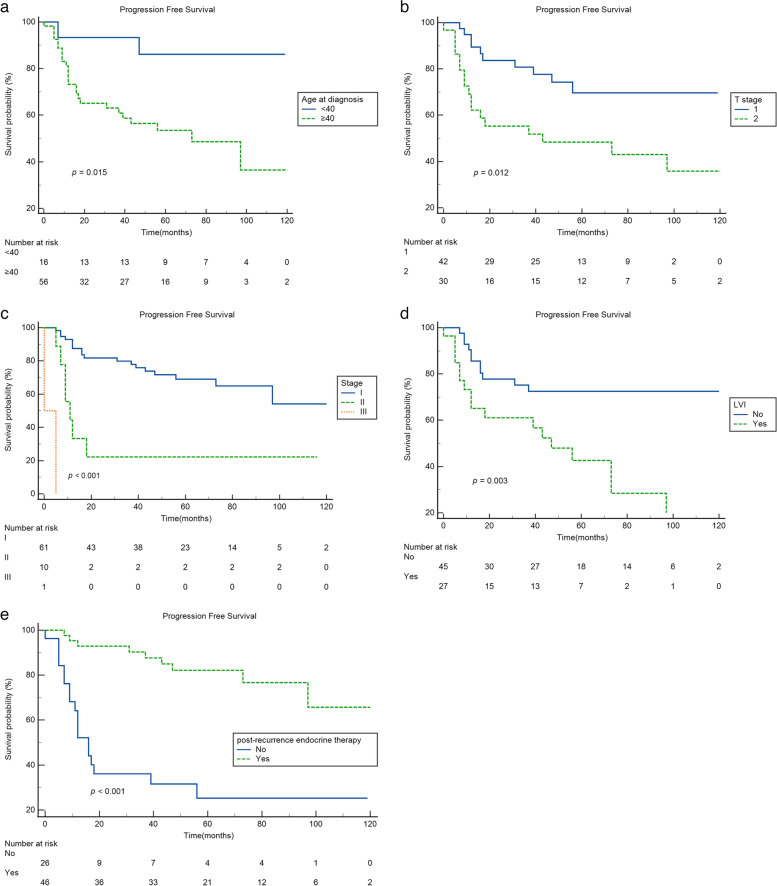
Post-recurrence progression free survival(P-PFS). **a** Age. **b** T stage. **c** Pathologic stage. **d** LVI. **e** Post-recurrence endocrine therapy

Kaplan–Meier curves of P-PFS according to age, T stage, pathologic stage, LVI, and post-recurrence endocrine therapy are presented in Fig. 1. Estimated 5-year P-PFS rates were 86.2%, 69.7%, 69.0%, 42.7%, and 82.2% for patients with age < 40 years at diagnosis (*p* = 0.015), patients with T1 stage (*p* = 0.012), patients with stage I (*p* < 0.001), patients with LVI (*p* = 0.003), and patients with post-recurrence endocrine therapy (*p* < 0.001), respectively.

In multivariate analysis, independent factors for progression were age at diagnosis, initial T stage, pathologic stage, LVI, and post-recurrence endocrine therapy. However, no difference was observed in site of first recurrence, number of first recurrence, post-recurrence chemotherapy, or post-recurrence radiotherapy (Table 3).

**Table 3:** Univariate and multivariate analysis of independent risk factors associated to progression after first ipsilateral recurrence

**Characteristics**	**No (*n* = 46) (%)**	**Yes (*n* = 27) (%)**	**Univariate analysis**		**Multivariate analysis**	
			**Crude HR (95%CI)**	***P* value**	**Adjusted HR (95%CI)**	***P* value**
Age (year) at diagnosis				0.031		0.027
< 40	14 (30.4)	2 (7.4)	ref		ref	
≥40	32 (69.6)	25 (92.6)	4.940 (1.162–20.998)		5.541 (1.216–25.250)	
Breast operation				0.815		
Breast conserving surgery	29 (63.0)	15 (55.6)	ref			
Mastectomy	17 (37.0)	12 (44.4)	1.095 (0.512–2.341)			
Axillary operation				0.056		
No	1 (2.2)	0	ref			
SLNB	20 (43.5)	4 (14.8)	3614.374 (0–8.176E114)			
ALND	25 (54.3)	23 (85.2)	13,308.201 (0–3.006E115)			
pT				0.017		0.046
1	32 (69.6)	10 (37.0)	ref		ref	
2	14 (30.4)	17 (63.0)	2.600 (1.184–5.709)		3.255 (1.022–10.363)	
pN				0.009		0.110
0	35 (76.1)	17 (63.0)	ref		ref	
1	8 (17.4)	7 (25.9)	1.382 (0.573–3.335)		0.975 (0.400–2.375)	
2	3 (6.5)	1 (3.7)	0.623 (0.082–4.712)		0.551 (0.061–4.962)	
3	0	2 (7.4)	59.942 (8.156–440.544)		14.851 (1.627–135.571)	
Pathologic stage				<0.001		0.022
I	43 (93.5)	18 (66.7)	ref		ref	
II	3 (6.5)	7 (25.9)	4.038 (1.652–9.871)		2.194 (0.823–5.854)	
III	0	2 (7.4)	80.273 (10.782–597.654)		20.642 (2.569–165.849)	
ER status				0.290		
Negative	1 (2.2)	1 (3.7)	ref			
Positive	45 (97.8)	26 (96.3)	0.333 (0.044–2.549)			
PR status				0.116		
Negative	8 (17.4)	7 (25.9)	ref			
Positive	38 (82.6)	20 (74.1)	0.498 (0.209–1.188)			
HG				0.076		
1	15 (32.6)	3 (11.1)	ref			
2	22 (47.8)	15 (55.6)	2.903 (0.838–10.059)			
3	9 (19.6)	9 (33.3)	3.844 (1.032–14.317)			
Ki-67				0.246		
≤15%	26 (56.5)	12 (44.4)	ref			
> 15	20 (43.5)	15 (55.6)	1.573 (0.732–3.378)			
LVI				0.013		0.048
No	33 (71.7)	11 (40.7)	ref		ref	
Yes	12 (26.1)	16 (59.3)	2.914 (1.346–6.305)		1.959 (0.863–4.447)	
Unknown	1(2.2)	0				
Adjuvant chemotherapy				0.307		
No	23 (50.0)	12 (44.4)	ref			
Yes	23 (50.0)	15 (55.6)	1.491 (0.693–3.206)			
Adjuvant radiotherapy				0.771		
No	16 (34.8)	12 (44.4)	ref			
Yes	30 (65.2)	15 (55.6)	0.893 (0.418–1.910)			
Adjuvant endocrine therapy				0.602		
No	2 (4.3)	1 (3.7)	ref			
Yes	44 (95.7)	26 (96.3)	1.704 (0.230–12.607)			
Site of first recurrence				0.150		
Breast	30 (65.2)	10 (37.0)	ref			
Axilla	7 (15.2)	7 (25.9)	2.114 (0.801–5.580)			
Chest wall/skin	7 (15.2)	7 (25.9)	2.037 (0.772–5.377)			
Two or more	2 (4.3)	3 (11.1)	3.840 (1.048–14.070)			
Number of recurrence				0.11		
One	44 (95.7)	24 (88.9)	ref			
Two or more	2 (4.3)	3 (11.1)	2.683 (0.801–8.993)			
Post-recurrence chemotherapy				0.125		
No	27 (58.7)	11 (40.7)	ref			
Yes	19 (41.3)	16 (59.3)	1.831 (0.846–3.964)			
Post-recurrence radiotherapy				0.651		
No	35 (76.1)	21 (77.8)	ref			
Yes	11 (23.9)	6 (22.2)	0.811 (0.327–2.012)			
Post-recurrence endocrine therapy				<0.001		<0.001
No	9 (19.6)	18 (66.7)	ref		ref	
Yes	37 (80.4)	9 (33.3)	0.173 (0.076–0.389)		0.176 (0.070–0.442)	

*SLNB* Sentinel lymph node biopsy, *ALND* Axillary lymph node dissection, *ER* Estrogen receptor, *PR* Progesterone receptor, *HG* Histologic grade, *LVI* Lymphovascular invasion

### Impact of IILRR on post-recurrence overall survival (P-OS)

During a median follow-up time from the first ipsilateral recurrence of 58.0 months (range: 1.0–171.0 months), 14 deaths were recorded. Factors significantly associated with P-OS in univariate analysis were initial N stage, pathologic stage, ER, PR, LVI, site of first recurrence, number of first recurrence, post-recurrence chemotherapy, and post-recurrence endocrine therapy (Table 4). Multivariate analysis showed that pathologic stage, post-recurrence endocrine therapy, and progression were independent factors associated with P-OS (Table 4). There was no statistical difference in the site of first recurrence (*p* = 0.476), number of first recurrences (*p* = 0.001), or post-recurrence chemotherapy (*p* = 0.190).

**Table 4:** Univariate and multivariate analysis of independent risk factors associated to survival after first ipsilateral recurrence

Characteristics	No (*n* = 59) (%)	Yes (*n* = 14) (%)	Univariate analysis		Multivariate analysis	
			Crude HR (95%CI)	*P* value	Adjusted HR (95%CI)	*P* value
Age (year) at diagnosis				0.392		
< 40	14	2	ref			
≥40	45	12	1.911 (0.423–8.633)			
Breast operation				0.297		
Breast conserving surgery	35	9	ref			
Mastectomy	24	5	0.535 (0.162–1.765)			
Axillary operation				0.674		
No	1	0	ref			
SLNB	21	3	5719.640 (0–8.406E165)			
ALND	37	11	10,285.682 (0–1.510E165)			
pT				0.094		
1	37	5	ref			
2	22	9	2.563 (0.853–7.701)			
pN				0.010		0.140
0	45	7	ref		ref	
1	11	4	1.297 (0.357–4.714)		0.273 (0.036–2.084)	
2	3	1	1.555 (0.190–12.745)		10.122 (0.399–256.539)	
3	0	2	87.290 (7.554–1023.339)		91.013 (6.209–1334.033)	
Pathologic stage				<0.001		<0.001
I	55	6	ref		ref	
II	4	6	12.378 (3.679–41.646)		11.051 (2.911–41.951)	
III	0	2	199.526 (15.719–2532.702)		113.398 (8.174–1573.235)	
ER status				0.045		0.604
Negative	1	1	ref		ref	
Positive	58	13	0.110 (0.013–0.950)		0.502 (0.037–6.794)	
PR status				0.002		0.348
Negative	9	6	ref		ref	
Positive	50	8	0.176 (0.058–0.532)		0.405 (0.061–2.676)	
HG				0.069		
1	17	1	ref			
2	30	7	3.522 (0.432–28.723)			
3	12	6	7.883 (0.934–66.539)			
Ki-67			0.063	0.076		
≤15%	34	4	ref			
> 15	25	10	2.872 (0.895–9.213)			
LVI				0.035		0.483
No	40	4	ref		ref	
Yes	18	10	4.085 (1.264–13.205)		2.744 (0.532–14.141)	
Unknown	1	0				
Adjuvant chemotherapy				0.158		
No	30	5	ref			
Yes	29	9	2.209 (0.736–6.633)			
Adjuvant radiotherapy				0.325		
No	23	5	ref			
Yes	36	9	1.821 (0.552–6.009)			
Adjuvant endocrine therapy			0.761	0.762		
No	2	1	ref			
Yes	57	13	0.730 (0.094–5.635)			
Site of first recurrence				0.049		0.476
Breast	35	5	ref		ref	
Axilla	11	3	1.661 (0.394–7.008)		1.118 (0.500–2.502)	
Chest wall or skin	11	3	1.462 (0.333–6.410)		1.651 (0.647–4.218)	
Two or more	2	3	7.395 (1.742–31.399)		3.327 (1.183–9.358)	
Number of recurrence				0.007		1.000
One	57	11	ref		ref	
Two or more	2	3	6.062 (1.631–22.526)		1.000 (0.057–17.511)	
Post-recurrence chemotherapy				0.005		0.190
No	36	2	ref		ref	
Yes	23	12	8.697 (1.920–39.399)		3.337 (0.551–20.204)	
Post-recurrence radiotherapy				0.223		
No	44	12	ref			
Yes	15	2	0.390 (0.086–1.770)			
Post-recurrence endocrine therapy				<0.001		<0.001
No	15	12	ref		ref	
Yes	44	2	0.069 (0.015–0.309)		0.080 (0.016–0.404)	
Progression				0.039		<0.001
No	46	0	ref		ref	
Yes	13	14	160.653 (1.278–20192.383)		143.561 (1.409–1850.352)	

*SLNB* Sentinel lymph node biopsy, *ALND* Axillary lymph node dissection, *ER* Estrogen receptor, *PR* Progesterone receptor, *HG* Histologic grade, *LVI* Lymphovascular invasion

**Fig. 2:**
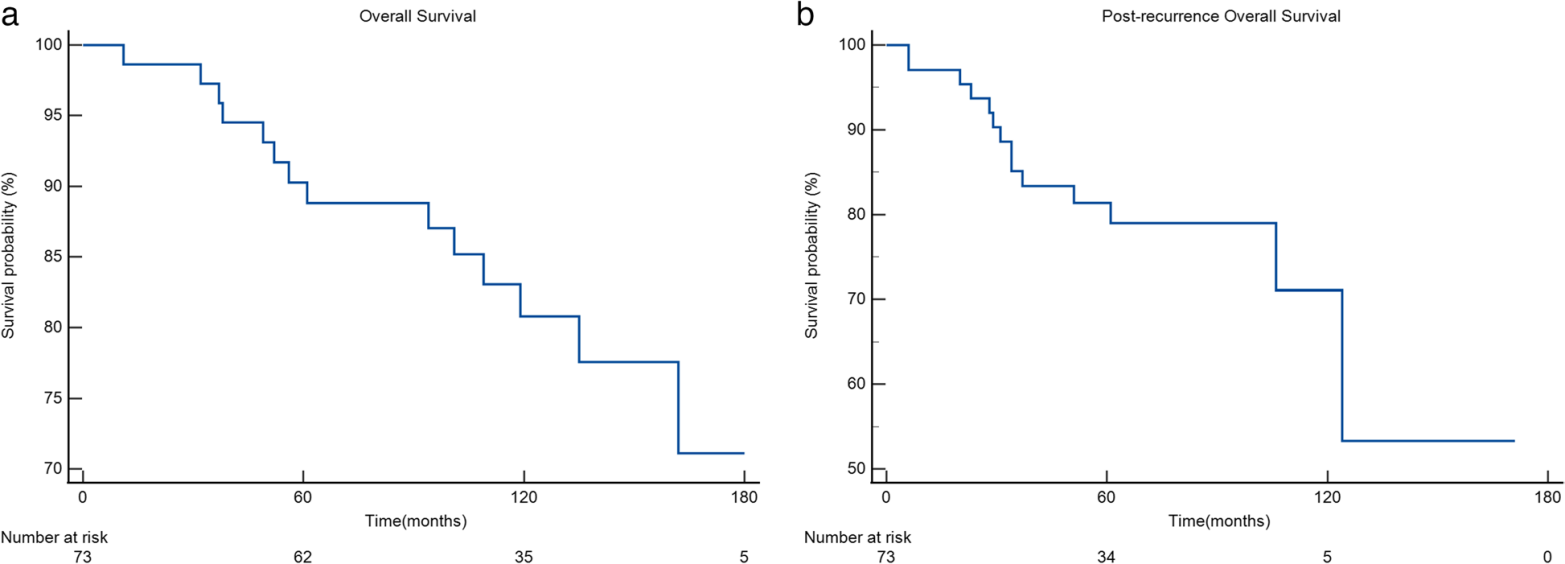
Overall survival(OS) and post-recurrence OS (P-OS)

**Fig. 3:**
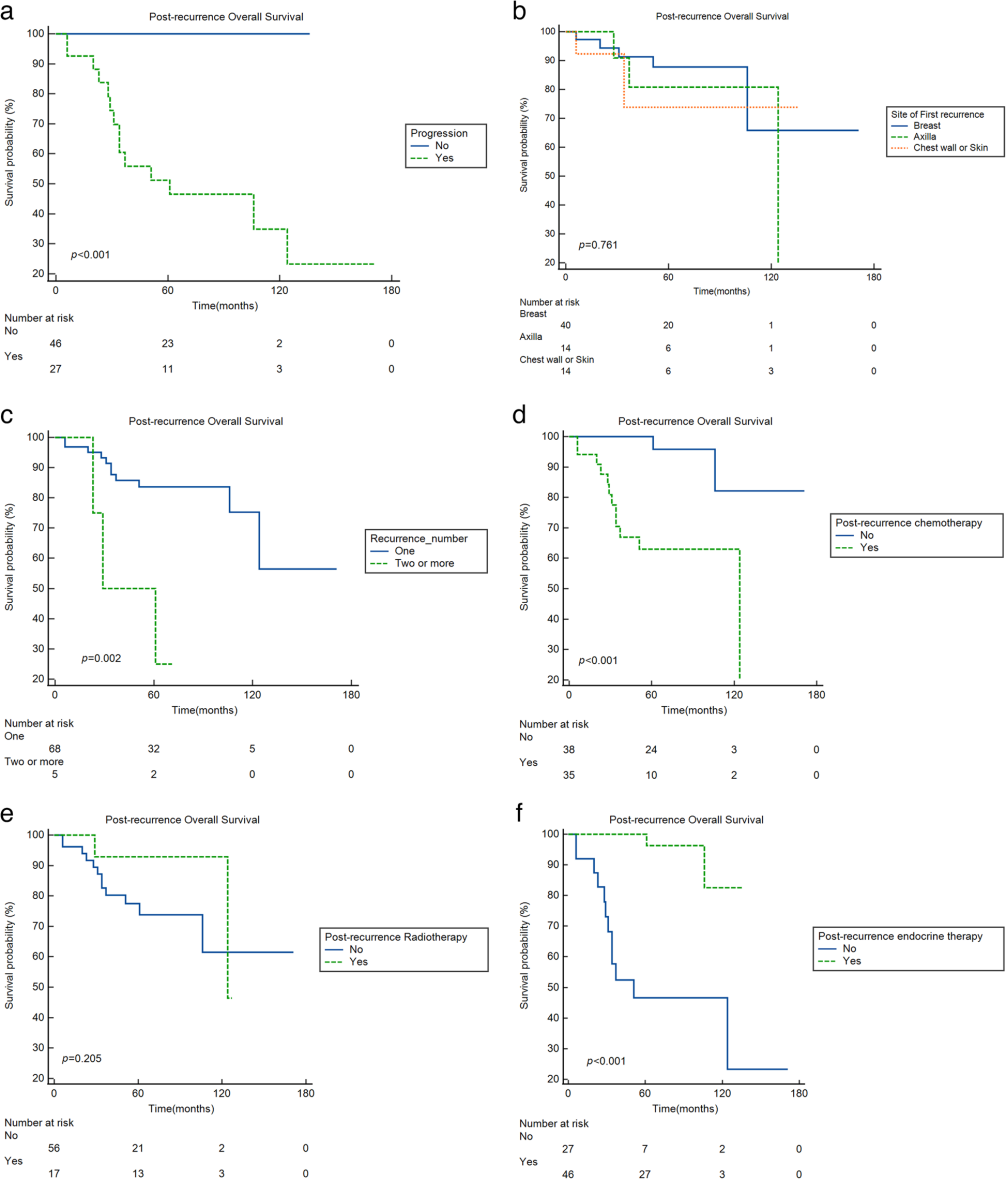
Post-recurrence overall survival (P-OS) according to progression, site of first recurrence, number of recurrence, and post-recurrence treatment. **a** Progression. **b** Site of first recurrence. **c** Number of recurrence. **d** Post-recurrence chemotherapy. **e** Post-recurrence radiotherapy. **f** Post-recurrence endocrine therapy

The 5/10-year Kaplan–Meier OS and P-OS for patients who underwent a post-recurrence treatment were 90.3%/80.8% and 81.4%/71.1%, respectively (Fig. 2). Kaplan–Meier curves of P-OS stratified according to progression, site of first recurrence, number of recurrences, and post-recurrence treatment are presented in Fig. 3. There were significant differences in P-OS according to presence or absence of progression (*p* < 0.001), number of first recurrences (*p* = 0.002), post-recurrence chemotherapy (*p* < 0.001), and post-recurrence endocrine therapy (*p* < 0.001). There was no difference otherwise.

## Discussion

In this large multi-center cohort of 3373 consecutive HER2-negative luminal breast cancer patients, we analyzed the rate of IILRR and outcome after IILRR. Few studies have focused on IILRR because advances in surgical technique, radiotherapy, and extensive use of adjuvant systemic therapies have reduced the rate of local recurrences in patients with HER2-negative luminal breast cancer than before [4, 8, 17]. Results of this study indicated that the incidence of IILRR of HER2-negative luminal breast cancer was 2.37%. Previous studies have suggested that the overall incidence of IILRR ranges from 5 to 10% after 10 years of follow-up [6, 18, 19]. In ABCSG8 trial including HR-positive breast cancer, the cumulative incidence of local recurrences was 2.6% at a median follow-up of 72.3 months [17]. In our study, only HER2-negative luminal breast cancer with good prognosis was investigated. The incidence was lower than previously reported incidence.

When examining the progression after IILRR, 27 (37%) out of 73 patients eventually relapsed after IILRR. Most (85.2%) of them eventually developed distant metastasis. This study confirmed that IILRR was associated with progression and eventually mortality. Our present findings are consistent with previous findings. Fisher et al. and Veronesi et al. have reported an adjusted relative risk of developing distant disease after ipsilateral breast tumor recurrence (IBTR) [18, 19]. Other studies have identified IBTR as an independent predictor of distant metastases and poor survival [20–22]. In National Surgical Adjuvant Breast and Bowel Project (NSABP) protocol, with or without node metastasis, patients with IBTR and other LRR showed significantly poorer prognosis [7, 8]. In a cohort of 2669 lumpectomy-treated patients in five protocols of node-positive disease, the relative hazard of distant disease after recurrence was 2.72 (95% CI: 2.23 to 3.33) and 5-year OS rates after IBTR and other LRR were 59.9% and 24.1%, respectively [7]. In a cohort of 3799 lumpectomy-treated patients with node-negative disease, the relative hazard of distant disease after recurrence was 3.41 (95% CI: 2.70 to 4.30) and 5-year OS rates after IBTR and other LRR were 76.6% and 34.9%, respectively [8]. Thus, IILRR is associated with a poor outcome, i.e., an increased risk of distant metastases and breast cancer related mortality [8, 9].

In our series, P-OS rates at 5 and 10 years were 81.4% and 71.1%, respectively. Other series have documented comparable survival rates, with 5-year OS ranging from 59 to 84% and 10-year OS ranging from 57 to 72% after IBTR [23–25]. The reason why 5- and 10-year survival rates after IILRR appeared to be higher than those of other studies might be because our analysis was conducted only on HR-positive and HER2-negative breast cancer patients with good prognosis. Most of the previous studies reported a worse prognosis for patients with local recurrence compared with those without local recurrence, whereas some studies found a similar prognosis for patients with and without local recurrence [9, 10, 26, 27]. In the present study, progression occurred only in 37% of patients with IILRR. However, progression itself was confirmed as a statistically significant factor for OS after IILRR. Thus, IILRR is ultimately associated with lower mortality.

In our study, factors affecting progression and survival after IILRR included age at first diagnosis, pathologic stage, and post-recurrence endocrine therapy. This suggests that clinicopathologic factors at the time of first diagnosis have a more important influence on the overall prognosis of patients than factors after IILRR. Our investigation of patient age at diagnosis as a prognostic factor found that age was an important risk factor for IILRR, but not for P-OS. Contrary to expectations, the site or number of IILRR was not associated with progression or post-recurrence survival. There have been several studies on factors affecting the prognosis after IILRR. Several clinicopathologic factors have been investigated as potential factors related to prognosis after local or locoregional recurrence [17]. According to several previous studies, in patients with HR-positive breast cancer, there were prognostic factors significantly associated with outcome following recurrences, including initial clinical and pathological characteristics such as grading, size, nodal involvement, age at diagnosis, and time to local recurrence [17, 28, 29]. The risk of developing distant metastases after locoregional recurrence or simultaneously having locoregional recurrence was higher in patients ≤ 45 years than in older patients (9% vs. 1%, *p* < 0.001), independently of other clinic-pathologic factors [17, 30]. Age at diagnosis is known to be an important predictor of survival as well as progression [7, 8]. In our study, age at diagnosis had an effect on progression. However, there was no difference in post-recurrence survival. This might be because the rate of receiving chemotherapy at progression was higher in younger patients. Similar to our results, Nottage et al. have found that age is an important risk factor for IBTR, but not for disease-specific survival [23].

Few studies have assessed the role of systemic treatments after surgical excision of local recurrence. The potential benefit of systemic treatments such as chemotherapy or endocrine therapy as therapy after local treatment of recurrence remains unclear, especially for HR-positive subtypes [17]. In a study of Waeber et al., tamoxifen significantly improved post-recurrence disease-free survival of HR-positive breast cancer patients after local treatment for IILRR, as it was 6.5 years with tamoxifen and 2.7 years with no treatment (*p* = 0.053) [31]. However, there was no statistically significant difference in overall survival (11.5 years vs. 11.2 years, *p* = 0.175) [31]. In a chemotherapy for isolated locoregional recurrence of breast cancer (CALOR) randomized trial, adjuvant chemotherapy after IILRR was significantly more effective for patients with ER-negative breast cancer (*p* = 0.046), but not for patients with ER-positive breast cancer (*p* = 0.43) [1]. In our results, post-recurrence chemotherapy or radiotherapy did not affect the progression or P-OS of HR-positive or HER2-negative breast cancer. Although the standard therapy for IILRR has not yet been established, based on the results of this study, endocrine therapy might play an important role in prognosis rather than chemotherapy or radiotherapy after IILRR.

Our study has several limitations. Because of the low incidence of IILRR, the number of analyzed patients is too small despite being a multi-center study. Also, it was not possible to analyze the difference according to the time when IILR occurred due to the small number of cases. Time to local recurrence is the most relevant prognostic factor for development of distant metastasis and poor prognosis after local recurrence [10]. Local recurrences occurring within 2–3 years from first diagnosis are generally associated to a worse distant disease-free survival and higher risk of evolving to metastatic disease, independently of hormone receptor status [8, 10, 28, 29]. Therefore, our findings may limit the generalizability of our findings to all HER2-negative luminal breast cancer patients with isolated ipsilateral local and regional recurrence. Also, because this study was conducted a single nationality (Korean women), our findings may limit the generalizability of our findings to other racial and ethnic groups. Nevertheless, the follow-up time is relatively long. Details of the recurrence including site and number of the recurrent lesion are completely collected. Since all patients underwent surgery after IILR, there was no surgical bias. Treatments of IILRR out of surgery such as systemic therapy or radiotherapy and their impact on survival are recorded or analyzed in this study. Therefore, further research with large number of patients with multi-center participation is required to evaluate prognostic factors after isolated ipsilateral local and regional recurrence in patients with HER2-negative luminal breast cancer.

## Conclusions

Age at diagnosis, pathologic stage at diagnosis, and post-recurrence endocrine therapy were associated with prognosis after IILRR in HER2-negative luminal breast cancer. There is no standard treatment after local resection of IILRR. Results of this study suggest that post-recurrence endocrine therapy might play an important role in prognosis rather than chemotherapy or radiotherapy in HER2-negative luminal breast cancer.

## Data Availability

The datasets used and/or analyzed during the current study are available from the corresponding author on reasonable request.

## Abbreviations

LRR: Locoregional recurrence
HR: Hormone receptor
IILRR: Ipsilateral local and regional recurrence
HER2: Human epidermal growth factor 2
ALND: Axillary lymph node dissection
SLNB: Sentinel lymph node biopsy
AJCC: American Joint Committee on Cancer
ER: Estrogen receptor
PR: Progesterone receptor
IHC: Immunohistochemistry
FISH: Fluorescence in situ hybridization
SISH: Silver in situ hybridization
DFS: Disease-free survival
P-PFS: Post-recurrence progression-free survival
P-OS: Post-recurrence overall survival
OS: Overall survival
HG: Histologic grade
LVI: Lymphovascular invasion
IBTR: Ipsilateral breast tumor recurrence
